# Multimatrix Composite Materials for Rocket Nozzle Manufacturing: A Comparative Review

**DOI:** 10.3390/polym17212946

**Published:** 2025-11-04

**Authors:** Mohammed Meiirbekov, Mukhammed Sadykov, Assem Kuandyk, Marat Nurguzhin, Marat Janikeyev, Partizan Gulmaira, Laura Mustafa, Nurmakhan Yesbolov

**Affiliations:** 1 JSC “National Center of Space Research and Technology”, Almaty 050010, Kazakhstan; a.kuandyk@spaceres.kz (A.K.); nurguzhin.m@spaceres.kz (M.N.); m.janikeyev@spaceres.kz (M.J.); g.partizan@spaceres.kz (P.G.); l.mustafa@spaceres.kz (L.M.); n.yesbolov@spaceres.kz (N.Y.); 2 Faculty of Mechanics and Mathematics, Al-Farabi Kazakh National University, Almaty 050040, Kazakhstan

**Keywords:** polymer matrix composites, nozzle, rocket, C/C, SiC/SiC, C/SiC, thermal resistance, modification

## Abstract

Rocket engine nozzle blocks operate under extreme thermal and oxidative loads, requiring materials with high temperature resistance, dimensional stability, and a predictable lifetime without active cooling. This review provides a comparative overview of multimatrix composite materials-including C/C, C/SiC, SiC/SiC, MMC, and polymer-based ablative systems-representing the full spectrum of materials used in non-cooled rocket nozzles. The study highlights the evolutionary continuum from polymeric ablative systems to carbon, ceramic, and metallic matrices, demonstrating how each class extends operational limits in temperature capability, reusability, and structural integrity. Polymer and ablative composites serve as the foundation of thermal protection through controlled ablation and insulation, while carbon- and ceramic-based systems ensure long-term performance at ultra-high temperatures (>1600 °C). MMCs bridge these classes by combining strength, impact toughness, and thermal conductivity in transition zones. Particular attention is given to manufacturing technologies such as PIP, CVI, LPI, RS, powder metallurgy, casting, diffusion bonding, and filament winding, emphasizing their effect on microstructure, porosity, and lifetime. A practical selection matrix linking nozzle zones, mission profiles, and composite types is proposed, outlining trade-offs among performance, mass, lifetime, and manufacturability, and guiding the design of next-generation thermal protection and propulsion systems based on the multimatrix concept.

## 1. Introduction

The nozzle block of a rocket engine is a key component that converts the thermal energy of combustion products into the directed kinetic energy of the exhaust jet and largely determines the specific impulse and service life of the propulsion system. The nozzle design typically includes a throat insert, a convergent-divergent section, a load-bearing shell, and attachment/thrust-vectoring units; all of these elements operate under extreme conditions of temperature (~3000 °C), oxidative environment, steep thermal gradients, and intense mechanical loads [1,2,3]. Under such conditions, conventional heat-resistant alloys (nickel-based, molybdenum-based, and others) rapidly lose their load-bearing capacity and become susceptible to embrittlement and corrosive wear. This has driven the transition toward composite materials with tailored thermal and erosion resistance, combined with reduced density [4,5].

**Figure 1:**
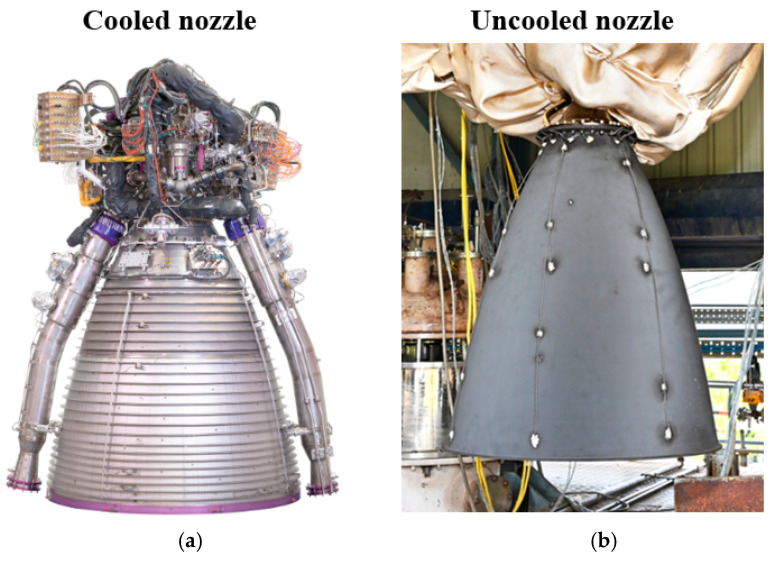
Classification of nozzle blocks: (**a**) cooled nozzle block of the Vulcain 2.1 rocket [6] (**b**) uncooled nozzle block of the rocket [7].

The primary factor determining the architecture of a nozzle block and the range of applicable materials is the method of heat removal. In rocket engineering practice, two main classes of nozzles have been established: uncooled and cooled (Figure 1).

Cooled nozzles employ active heat removal-regenerative, film, or transpiration cooling. The liners of cooling channels are typically manufactured from high-thermal-conductivity copper alloys (CuCrZr, GRCop-84/42), while the load-bearing shell is made of nickel-based superalloys (Inconel 718/625), titanium alloys, or corrosion-resistant steels; hybrid designs such as a copper liner combined with an external composite overwrap are also common. In the throat region of such nozzles, inserts made of graphite, C/C, or C/SiC are often used to extend service life [8,9,10,11,12].

Uncooled nozzles maintain thermal balance through radiative heat transfer and/or controlled ablation. Materials employed include graphite and carbon–carbon (C/C) composites for throat inserts and liners; ablative composites based on phenolic resins (carbon-/silica-phenolic); carbon–ceramic systems (C/SiC); and, for ultra-high-temperature applications, ultra-high-temperature ceramics (UHTCs) such as ZrB_2_-SiC and HfB_2_-SiC [13,14,15,16].

The preference for uncooled composite nozzles is driven by their ability to withstand extreme temperatures while maintaining relatively low mass, high thermal resistance, and erosion durability. The structural simplicity enabled by the absence of cooling circuits ensures reliability, mass reduction, and manufacturing efficiency, making such nozzles particularly attractive for solid rocket motors in expendable applications [2,17,18].

**Figure 2:**
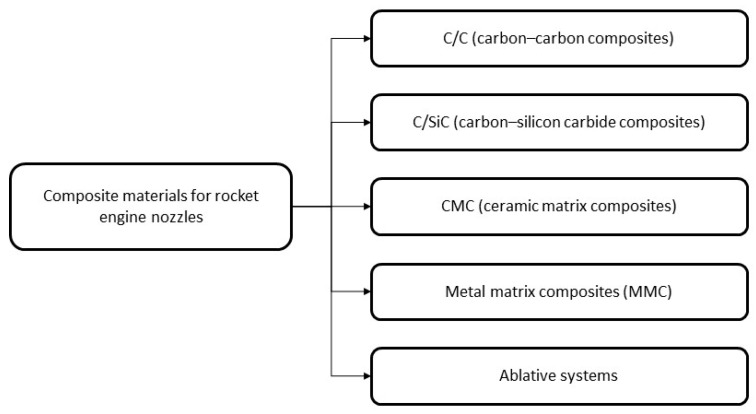
Classification of composite materials for rocket engine nozzles.

The wide variety of reinforcement architectures, matrix types, and synthesis methods complicates the selection of a universal material, necessitating a systematic classification that accounts for thermal stability, structural requirements, and manufacturing constraints. Based on an analysis of current research, patented solutions, and existing technologies, five principal classes of composite materials employed in nozzle blocks can be distinguished. They differ in matrix type, reinforcement approach, operating temperature range, and thermal protection mechanisms. A structural classification of these materials is presented in Figure 2.

Each of the mentioned groups of composites is distinguished by a specific balance of thermal resistance, strength, and manufacturability, with the processing technology playing a decisive role. C/C remain stable at temperatures above 2500 °C and are produced by techniques such as polymer infiltration and pyrolysis (PIP), chemical vapor infiltration (CVI), liquid-phase infiltration (LPI), or their combinations [19,20,21,22,23]. C/SiC composites, suitable for operation up to 1300 °C, are produced using liquid-phase infiltration (LPI), chemical vapor infiltration of silicon carbide (CVI), impregnation with polycarbosilanes followed by pyrolysis (PIP), and reaction sintering (RS) [24,25,26,27,28]. Ceramic matrix composites (SiC/SiC), providing thermal stability above 1600 °C, are fabricated using CVI, PIP, LPI, and hot sintering methods, which enable high density and oxidation resistance. Metal matrix composites (MMC), operating up to 1000 °C, are manufactured by powder metallurgy, liquid-phase infiltration (LPI), casting, diffusion bonding, and spray deposition techniques [14,29,30,31,32,33,34,35]. Ablative composites are fabricated by compression molding, filament winding, and 3D molding followed by sintering or vulcanization, providing thermal protection through controlled surface degradation under extreme temperatures [13,36,37,38].

The industrial applicability of the aforementioned solutions is confirmed by examples from modern rocket programs. For instance, the RL10B-2 engine (Aerojet Rocketdyne, Sacramento, CA, USA) employs C/C composites with SiC coatings. The nozzle of the Vinci engine (Ariane Group, Vernon, France) utilizes a C/SiC composite, combining high strength with low weight. Graphite and C/SiC nozzles are also applied in the HM7B engine. In NASA’s X-33 program (USA), successful tests of SiC/SiC composites were carried out under liquid hydrogen rocket engine conditions, demonstrating their potential for reusable systems [6,39,40].

The selection of a composite material for a rocket nozzle block requires consideration not only of thermal resistance but also of numerous interrelated factors-reinforcement architecture, fabrication technology, oxidation resistance, mechanical strength, ability to withstand ablative loads, and manufacturing scalability. The review presented in this article of five key classes of composite materials-from carbon–carbon and carbon–ceramic to metal matrix and ablative systems-makes it possible to systematize their characteristics, compare advantages and limitations, and define rational application zones within nozzle designs. A comprehensive analysis of these materials is essential for the development of next-generation thermal protection solutions capable of ensuring the reliability and durability of rocket engines under extreme thermomechanical conditions.

The aim of this article is to review and synthesize modern technologies for manufacturing nozzle blocks from composite materials, to evaluate their performance under extreme thermomechanical loads, and to formulate recommendations for material and process selection for practical implementation.

## 2. Properties and Characteristics of Different Types of Composites for Rocket Nozzle Assembly

**Figure 3:**
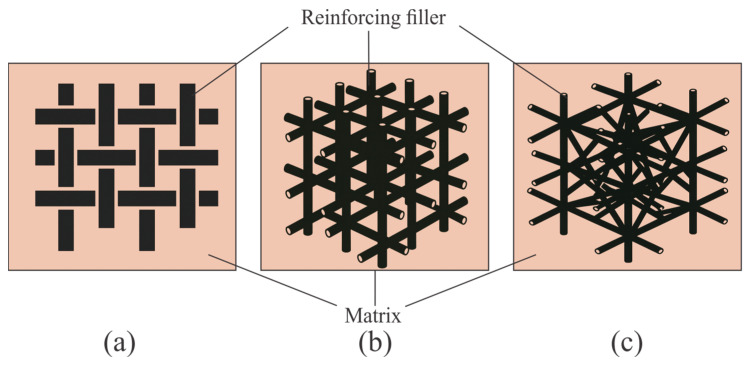
Schematic representation of reinforced composites: (**a**) 2D reinforcement, (**b**) 3D reinforcement, (**c**) 4D reinforcement.

The rational selection of materials for rocket engine nozzles requires a comprehensive analysis of their behavior under high temperatures, oxidative environments, and mechanical loads. Modern thermal protection composites are characterized by diverse reinforcement architectures-ranging from two-dimensional (2D) to three- and four-dimensional (3D/4D) structures-that provide an optimal balance between strength and thermal resistance (Figure 3).

To produce thermal protection composites, widely used methods such as CVI, PIP, LPI, and pressing are employed, each offering specific advantages depending on the intended application. In this section, for each class of composite materials, the architectural features, fabrication methods, strength characteristics under high-temperature loads, as well as practical aspects of their application in rocket engine nozzle blocks are consistently examined.

### 2.1. Carbon–Carbon Composites (C/C)

Carbon–carbon composites (C/C) are materials consisting of carbon fibers embedded in a carbon matrix. They are distinguished by their exceptional thermal resistance (up to 3000 °C in inert environments, 1800–2000 °C in oxidative conditions), low coefficient of thermal expansion (0.5–2.0 × 10^−6^ K^−1^), and high tolerance to thermocyclic loading (demonstrate high resistance to multiple thermal cycles at ΔT ≈ 1000 °C). Owing to these properties, C/C composites are widely used in the aerospace sector, particularly in rocket engine nozzle assemblies [41,42,43].

The reinforcement architecture of C/C composites has a critical impact on their mechanical and thermal performance. In the reviewed literature, the primary focus is placed on 2D and 3D structures, while 4D reinforcement is considered a promising direction. As illustrated in Figure 3b, 2D architectures are formed through the lamination of woven fiber plies and are widely employed in thermally loaded components, despite their limited through-thickness strength (typically <20–30 MPa) [43,44,45,46]. 3D reinforcement, which provides spatial interconnection of fibers, demonstrates improved impact resistance (by 30–40% compared to 2D analogs) and enhanced delamination resistance [21,41,42,47]. 4D configurations, which incorporate variable fiber orientation and reinforcement density, are highlighted as technologically advanced solutions aimed at enhancing reliability under extreme thermal loading conditions (ΔT up to 1500–2000 °C). The fiber volume fraction in C/C composites typically ranges from 45 to 60 vol.%, depending on the fabric architecture and densification process used. This ratio provides an optimal balance between density, thermal conductivity, and mechanical integrity under thermal shock conditions [44,47].

**Figure 4:**
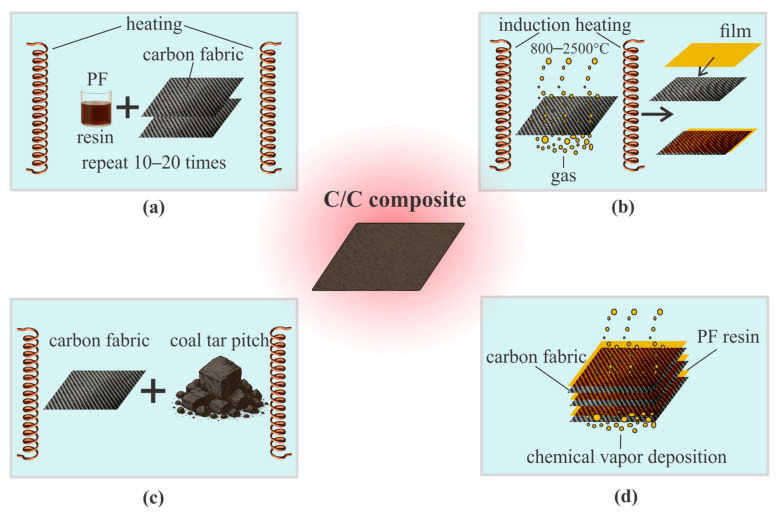
Methods for the fabrication of C/C composites: (**a**) PIP; (**b**) CVI; (**c**) LPI; (**d**) hybrid PIP + CVI.

The synthesis of C/C composites is carried out using a range of industrial technologies that differ in the type of precursors, the mechanism of carbonization, and the conditions for structural densification. The most widely employed methods include PIP, CVI, LPI, as well as their hybrid modifications (e.g., PIP + CVI) (Figure 4).

Figure 4a illustrates a schematic representation of the C/C composite fabrication process using the PIP method. This approach is widely employed for the formation of the carbon matrix through repeated impregnation of reinforcing structures with phenol-formaldehyde or furan resins, followed by pyrolysis in an inert atmosphere. As noted by several researchers [21,22,43,44,46,48], the repeated application of processing cycles (typically 5–10) enables effective structural densification, reduction in porosity from 20–25% to 8–12%, and enhancement of the material’s thermal and mechanical stability. The main advantages of this method include its technological simplicity, the wide availability of resins, and the ability to produce components with high density (1.75–1.90 g/cm^3^), homogeneous microstructure, and stable properties at temperatures exceeding 2000 °C.

Figure 4b shows the fabrication process of a C/C composite using the Chemical Vapor Infiltration (CVI) method. This process is based on the deposition of pyrolytic carbon from hydrocarbon gases (e.g., methane or propane) into a heated porous preform. The authors of several studies [21,22,41,44,46,48] noted that CVI enables the formation of a homogeneous carbon matrix with low porosity (5–10%) and minimal thermal defects. The authors of [48] employed CVI with a methane–propane mixture at 950 °C for 100 h. Other studies [21,44] report a typical temperature range of 900–1100 °C with processing durations of up to 200 h. Carbon deposition occurs through gas pyrolysis within the pores of the fibrous preform, providing uniform densification with final densities of 1.70–1.85 g/cm^3^ [22,46]. The main drawback of the method remains its long processing time (100–300 h for thick sections), as emphasized in [41]. Nevertheless, for critical components such as rocket engine nozzle blocks, CVI remains a reliable technique for producing thermally resistant C/C composites with high structural homogeneity.

Figure 4c presents a schematic of the C/C composite fabrication process using the Liquid Polymer Infiltration (LPI) method, in which the reinforcing structure is impregnated with molten resin or pitch. As noted in several studies [21,22,41,44,46], after impregnation, curing and pyrolysis are performed, and the cycle is repeated until the desired density is achieved. LPI is carried out at temperatures around 250–400 °C for resin or pitch infiltration, followed by pyrolysis at 800–1000 °C. As demonstrated in [21], infiltration can be performed under pressure (1–5 MPa) to enhance resin penetration into the pores of the preform. Compared to CVI, the LPI method features shorter processing cycles (tens of hours vs. hundreds of hours) and higher densification rates (achieving 1.65–1.80 g/cm^3^ after 4–6 cycles), making it attractive for large-scale production [22]. However, it is accompanied by shrinkage of 5–10% and cracking during carbonization, necessitating precise control of the temperature profile [41].

Figure 4d illustrates the fabrication of a C/C composite using a hybrid infiltration method that combines PIP and CVI stages. This approach allows the advantages of both technologies to be leveraged: the high structural homogeneity achieved during the CVI stage and the further increase in final density through subsequent PIP processing [21,41,44]. As noted in several studies, the initial densification using CVI ensures uniform deposition of pyrolytic carbon and minimizes macroporosity (<5%) [44], This is followed by additional resin infiltration (typically phenol-formaldehyde) with subsequent pyrolysis in PIP cycles, which significantly increases the final density of the composite up to 1.85–1.95 g/cm^3^ [21]. This hybrid scheme also allows the process to be adapted to complex component geometries and specific features of the fiber architecture [41].

The methods for forming the carbon matrix in C/C composites-including PIP, CVI, LPI, and their combinations-differ significantly in terms of processing time, complexity, and resulting structure. The choice of technology depends on the required density, homogeneity, allowable porosity, and component geometry. In some cases, hybrid approaches (e.g., PIP + CVI) enable an optimal balance between low porosity (5–7%) and manufacturing efficiency. However, regardless of the method employed, the resulting composites must undergo mandatory mechanical testing, including tensile, compressive, and bending tests. The averaged mechanical properties of C/C composites obtained from multiple literature sources are summarized in Table 1. The dataset was harmonized by annotating the testing atmosphere, exposure regime, and fabrication route, allowing direct comparison of performance across different processing techniques.

Table 1 summarizes the mechanical behavior of C/C composites under various loading conditions. The results indicate that the mechanical performance is primarily governed by reinforcement architecture, porosity level, and matrix densification route. Composites with 3D reinforcement demonstrate the highest structural efficiency due to improved fiber–matrix bonding and reduced defect density. The combination of CVI and PIP processing provides a denser and more uniform matrix, contributing to enhanced strength and fatigue resistance. In contrast, materials with higher residual porosity exhibit lower elastic response and reduced long-term stability. Overall, the dataset confirms the critical role of hybrid processing and 3D architectures in achieving balanced strength, stiffness, and thermal tolerance for C/C composites.

The use of C/C composites in structural components of rocket engines has been confirmed in several studies focusing on realistic operating conditions. In these works [15,23,60], experimental data are presented on the thermal protection and mechanical performance of C/C composites used in throat inserts and extendable nozzles of solid rocket motors, with a focus on thermal resistance, ablation resistance, and shape stability under cyclic thermal loading. Additionally, ref. [61] a probabilistic reliability analysis of a C/C composite nozzle assembly was conducted, taking into account thermomechanical fluctuations, demonstrating the applicability of such materials in the design of critically loaded components. In these studies, refs. [56,57,58] the features of oxidation resistance and high-temperature strength of various C/C composite architectures were examined, confirming their suitability under high-temperature gas flow and aggressive environments. Taken together, these data indicate the high maturity and practical relevance of C/C composites as thermally loaded structural materials for rocket engine nozzle assemblies.

### 2.2. Carbon–Ceramic Composites (C/SiC)

Carbon–ceramic composites (C/SiC) are promising materials for thermal protection systems of solid rocket motors (SRMs), particularly in nozzle assemblies, where high thermal resistance must be combined with mechanical strength. They combine the light weight and thermal conductivity of carbon fibers (λ ≈ 20–40 W·m^−1^·K^−1^ along fibers) with the high-temperature resistance of a ceramic matrix, providing stability at temperatures above 1600 °C (short-term up to 1800–2000 °C), good ablation resistance (erosion rates typically 0.01–0.03 mm/s), and structural stability in oxidative environments. The use of C/SiC composites enhances the reliability of rocket engines under repeated heating and cooling cycles (>500 cycles at ΔT ~1000 °C) and reduces the mass of structures by 20–30% compared to conventional metallic or ceramic components [24,25,62].

An analysis of the literature shows that in the development of C/SiC composites, the greatest attention is paid to fiber preforms with 2D and 2.5D architectures, which are widely used due to their technological accessibility and reproducibility of properties [25,26,63]. At the same time, in recent years there has been a consistent trend towards the use of 3D architectures, which provide higher thermomechanical stability (tensile strength 180–220 MPa vs. 120–160 MPa for 2D) and enhanced resistance to delamination (interlaminar shear strength increased by 40–50%) [64,65]. A separate direction involves hybrid systems (e.g., Z-pin reinforcement), aimed at strengthening the through-thickness direction (shear strength 25–30%) and stabilizing the protective layers during service. The fiber reinforcement fraction in C-SiC composites generally lies between 40 and 55 vol.%, slightly lower than that in C/C systems because part of the carbon matrix is converted into silicon carbide during infiltration [26] (Figure 3a,b).

**Figure 5:**
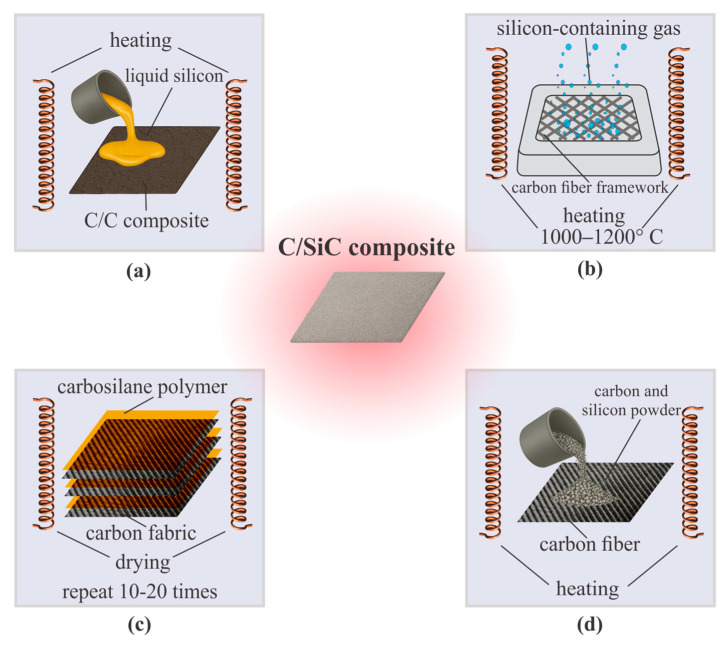
Methods for the fabrication of C/SiC composites: (**a**) LPI; (**b**) CVI; (**c**) PIP; (**d**) Reactive sintering.

Several industrial and laboratory approaches are used for the synthesis of C/SiC composites, differing in the mechanism of ceramic matrix formation, temperature regimes, and the number of processing cycles [24,66]. Figure 5 presents the main methods, including LPI, CVI, PIP, and RS, each of which has its own advantages and limitations in terms of strength, thermal resistance, and manufacturing cost.

In a number of studies [16,67,68] the LPI is considered one of the key industrial approaches for producing C/SiC composites. This method is based on impregnating a reinforcing fibrous preform with solutions or melts of polysiloxanes, polycarbosilanes, or other precursors, followed by heat treatment in an inert atmosphere, which leads to the formation of a ceramic matrix, e.g., SiC-based. Multiple cycles of infiltration and pyrolysis (typically 4–8 cycles) densify the structure, reduce porosity from 20–25% to 10–12%, and enhance thermomechanical stability. According to [68], such composites exhibit tensile strength in the range of 150–200 MPa and elastic modulus of 30–40 GPa, while [16] emphasizes their high reproducibility and processing flexibility. The LPI process scheme is shown in Figure 5a.

Figure 5b shows the sequence of stages of the CVI method for forming a dense and uniform ceramic matrix within a reinforcing carbon preform. Several studies have described CVI in detail as one of the key industrial approaches for producing C/SiC composites, particularly those used under high-temperature and oxidative conditions. According to [16,67,69,70,71] the method is based on impregnating a porous carbon preform with gaseous silicon-containing precursors, followed by their pyrolytic decomposition at temperatures around 1000–1200 °C, resulting in the deposition of silicon carbide within the pores and on the surface of the reinforcing fibers. The authors of [68,72] demonstrated that C/SiC composites produced via CVI exhibit higher tensile strength (200–250 MPa) and superior thermo-oxidative resistance (stable up to 1700 °C in air with protective coatings) compared to materials fabricated using alternative methods. Final densities typically reach 2.0–2.1 g/cm^3^ with residual porosity below 8%.

Figure 5c illustrates the process of fabricating C/SiC composites using the PIP method, which involves the sequential impregnation of a porous preform with a ceramic precursor (typically polycarbosilane), followed by heat treatment in an inert atmosphere to convert the polymer into a ceramic phase, and repetition of the cycle until the desired density is achieved and residual porosity is minimized. This processing route has been widely employed in several studies, including [63,65,67,73]. The consecutive infiltration-pyrolysis cycles (5–10 cycles) ensure the formation of a uniform ceramic matrix throughout the reinforcing structure while preserving the fiber architecture. Typical properties of PIP-derived C/SiC include tensile strength 160–210 MPa, modulus 25–35 GPa, and porosity 10–15%. These characteristics are critical for achieving high thermal resistance and mechanical strength under the thermal and mechanical loads typical of aerospace applications.

The RS method is widely used for the fabrication of C/SiC composites, providing high density and heat resistance within a relatively short processing time. According to the scheme shown in Figure 5d, the process involves the preliminary forming and densification of a carbon preform, followed by infiltration with liquid silicon and subsequent carburation at temperatures typically above 1450–1700 °C, resulting in the formation of a silicon carbide matrix with a controlled fraction of residual free silicon (5–15%). Studies have shown that the configuration of the fiber reinforcement and the parameters of thermal treatment determine the distribution of the SiC phase, porosity (often <5–7%), and the mechanical properties of the final material [24,26,27,28,63,74,75,76]. In particular, refs. [24,28,76] detail the stages of infiltration and reactive interaction, including methods to reduce residual free Si to improve strength (up to 250–300 MPa) and elastic modulus (40–50 GPa); Refs. [26,27,63] address the scaling of the technology and its influence on thermal resistance and oxidative stability; while [75,77] describe modifications of the starting raw materials to adapt the process to various operational conditions.

The C/SiC composite fabrication technologies discussed-LPI, CVI, PIP, and reactive sintering-represent complementary approaches, each with distinct technological advantages, limitations, and optimal application areas. The choice of a specific method is determined by requirements for matrix density (1.9–2.2 g/cm^3^) and uniformity, allowable residual porosity (typically <10%), thermo-oxidative stability (stable up to 1600–1800 °C depending on coatings), as well as economic and production considerations. Reactive sintering holds a special position as a high-throughput technique, enabling the achievement of high density and strength within a relatively short cycle (tens of hours vs. hundreds for CVI), whereas LPI, CVI, and PIP provide more precise control over phase distribution and the structure of the reinforcing framework. Overall analysis indicates that optimizing the combination of reinforcement architecture, precursor composition, and processing parameters can achieve the desired balance between mechanical strength, thermal resistance, and material durability.

The mechanical behavior of C/SiC composites was analyzed to assess their suitability for non-cooled nozzle components. The results, summarized in Table 2, have been harmonized by specifying the testing atmosphere, exposure mode, and fabrication route (RS, PIP, CVI). Such normalization enables a consistent comparison of performance across different processing techniques, highlighting how manufacturing route and environmental conditions affect the resulting mechanical properties.

Table 2 summarizes the mechanical performance of C/SiC composites under different loading conditions. The data show that their behavior is strongly influenced by reinforcement architecture, matrix density, and residual silicon content. Composites with optimized fiber–matrix interfaces and reduced porosity exhibit markedly higher strength and reliability, while excessive free silicon or incomplete carburation leads to premature degradation. The combination of CVI and reactive sintering routes provides superior densification and enhances toughness without compromising stiffness. Owing to the inherent anisotropy of fiber reinforcement, the longitudinal elastic modulus significantly exceeds the transverse one, defining the directional mechanical response of these materials. Overall, the dataset confirms that the balance between matrix consolidation and fiber bonding is the key factor controlling the performance and thermal stability of C/SiC composites in high-temperature environments.

Analysis of the literature indicates that C/SiC composites possess a balanced set of mechanical properties, ensuring their reliability under the thermo-mechanical and impact loads characteristic of rocket engine nozzle assemblies. These materials maintain stiffness and load-bearing capacity at elevated temperatures, exhibit resistance to dynamic effects, and provide long-term performance under cyclic loading conditions. Optimization of reinforcement architecture and fabrication techniques enables the achievement of the required combination of strength, thermal resistance, and durability, making C/SiC composites promising for use in critically loaded components of aerospace systems. According to [67], these composites are effectively employed as thermal protection elements and structural components of spacecraft, maintaining mechanical integrity under heat fluxes exceeding 1 MW·m^−2^. Studies [72,81] demonstrate the suitability of C/SiC composites for combustion chambers and rocket engine nozzle assemblies due to their ablation resistance and high fatigue strength under cyclic thermo-mechanical loading. Work [82] describes their application in membrane systems for aggressive and high-temperature environments, while [83]-reports their use in reusable thermal protection panels for spacecraft. Collectively, these studies confirm that C/SiC composites are a key material for components operating under the extreme conditions of aerospace applications.

### 2.3. Ceramic Matrix Composites (SiC/SiC)

Ceramic matrix composites (SiC/SiC composites) are high-temperature structural materials optimized for operation under intense thermal and mechanical loads. The uniformity of the reinforcing fibers and the SiC matrix ensures compatible thermal expansion (difference not exceeding 0.5–1.0 × 10^−6^ K^−1^) and reduces interfacial stresses. These composites retain their mechanical properties at temperatures above 1600–1700 °C, and exhibit resistance to oxidation in air up to 1500 °C for 100–200 h, erosion under heat fluxes of 1–5 MW·m^−2^, and cyclic loading for more than 10^3^–10^4^ cycles without catastrophic failure. Typical tensile strength values are in the range of 200–400 MPa, flexural strength 300–600 MPa, and fracture toughness 6–10 MPa·m^1/2^, while density reaches 2.4–3.0 g/cm^3^ with residual porosity of 5–15% [84,85,86]. These characteristics make SiC/SiC composites highly suitable for nozzle throats, diverging sections, thermal protection screens, and liners of SRM combustion chambers, where long-term durability under extreme heat fluxes exceeding 1–5 MW·m^−2^ is critical [84,85,86].

In the analyzed publications on SiC/SiC composites, the greatest attention is given to the architecture of the reinforcing preforms. Reviews [84,85,86,87,88] primarily focus on 2D and 3D textile structures, comparing their advantages and limitations (Figure 3a,b). Studies [89,90,91] emphasize the role of the interphase layer (most often pyrolytic carbon or BN with thickness 0.1–0.5 µm) and its interaction with the chosen reinforcement architecture. All authors agree that the type of fiber preform and the configuration of the interface largely determine the thermo-mechanical behavior of SiC/SiC composites and establish the balance between strength, fracture toughness, and high-temperature stability. The fiber volume fraction in SiC-SiC composites is usually within 30–45 vol.%, as the high matrix density and multiple impregnation cycles limit the achievable reinforcement content [84,85,86].

**Figure 6:**
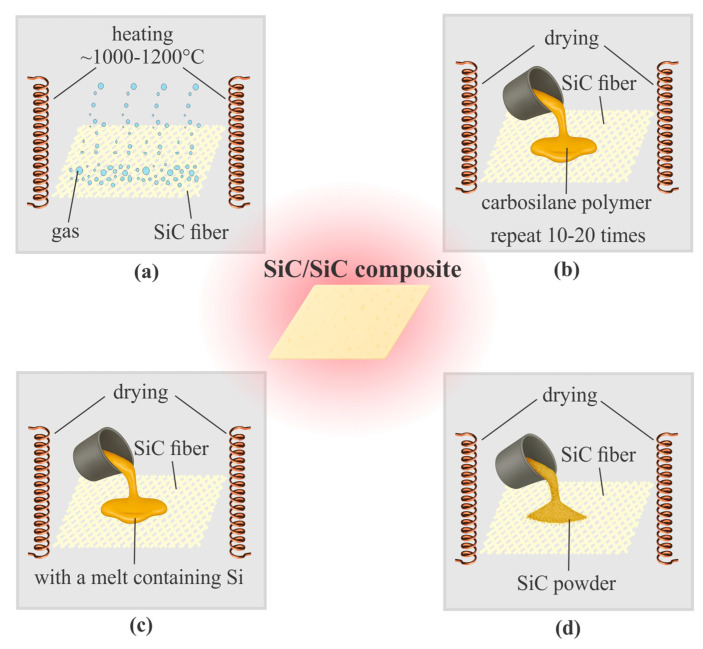
SiC/SiC fabrication schemes: (**a**) CVI; (**b**) PIP; (**c**) LPI; (**d**) Sintering.

Technologically, 2D architectures are implemented via slip infiltration with specified stacking sequences, whereas 3D architectures are formed using multi-axial winding and woven techniques for axisymmetric components. Regarding matrix fabrication methods, it should be noted that the choice of technology determines the density (2.3–3.0 g/cm^3^), defect content (residual porosity 5–20%), and thermal resistance of the final material. The most common processes include CVI, PIP, LPI, and sintering of powder preforms (Figure 6).

Figure 6a illustrates the CVI method, which is based on infiltrating a porous fiber preform with gaseous precursors (e.g., methyl-trichlorosilane, MTS) under reduced pressure or in a controlled inert gas atmosphere. The process is conducted at temperatures of 1000–1200 °C for durations up to 100–300 h, enabling thermal decomposition of the precursor and deposition of silicon carbide on the fiber surfaces and within the pores. Controlling the pressure (typically 1–10 kPa), temperature, gas composition, and flow rate allows the formation of a matrix with a tailored microstructure, residual porosity below 10%, and compatibility with the architecture of the reinforcing preform. Various modes-such as isothermal, temperature-gradient, and accelerated-determine the densification rate, defect distribution, and interphase layer characteristics. Several authors, including [84,92] highlight CVI as the industrial standard for forming the SiC matrix in SiC/SiC composites, emphasizing its ability to provide uniform fiber coating, minimize thermal stresses, and achieve high oxidation resistance at temperatures above 1400 °C. Reviews [84,92] detail the matrix formation mechanisms, the influence of process parameters on density and microstructure, as well as examples of applications for 2D and 3D textile architectures and turbine components. It is noted that optimization of the CVI regime is critical for long-term property stability and for enhancing fracture toughness under extreme heat flux conditions.

The PIP method is one of the most widely used technologies for matrix formation in SiC/SiC composites, schematically shown in Figure 6b. The process involves multiple cycles (10–20) of impregnating the reinforcing preform with a solution or melt of a polymeric precursor (e.g., polycarbosilanes or polysiloxanes), curing, and subsequent pyrolysis in an inert or reactive atmosphere at temperatures of 800–1200 °C, leading to the formation of a ceramic phase [93,94]. Since volumetric shrinkage during pyrolysis reaches 20–30% and leads to pore formation, multiple cycles of infiltration and heat treatment are typically required to achieve a density of 2.3–2.6 g/cm^3^ and residual porosity < 15% [85,86,95]. According to [84,96], the PIP method provides flexibility in designing the architecture of reinforcing structures (2D and 3D textile preforms) and enables targeted alloying of the matrix with additives such as B, Ti, Hf, or Zr, which improves mechanical properties and oxidation resistance by 20–40%. PIP technology represents an industrially mature solution, combining relative simplicity of equipment with the ability to produce matrices of complex chemical composition while preserving fiber integrity and minimizing interfacial damage.

The LPI method, schematically shown in Figure 6c, belongs to capillary-driven matrix fabrication techniques for SiC/SiC composites. In several studies [87,97,98,99], this method is implemented by impregnating a porous preform with silicon-based melts or resins, followed by carbonization and pyrolysis. The process is carried out at approximately 250–400 °C for binder introduction and 800–1000 °C for its decomposition, with the infiltration and heat treatment cycle repeated 5–10 times until the desired density is achieved. The LPI method enables deep infiltration and the formation of a dense matrix even in complex architectures, typically reaching 2.5–2.7 g/cm^3^ density with residual free silicon of 5–15%, making it both productive and adaptable for modifications and hybrid schemes. However, it offers limited control over microstructure, is susceptible to defects such as microcracks and incomplete wetting, and requires sophisticated equipment to handle reactive melts.

The sintering method, schematically shown in Figure 6d, is based on introducing SiC powder into the reinforcing preform, followed by drying and high-temperature treatment. During heating at 1800–2200 °C, the powder particles densify and bond, forming a matrix around the fibers. This approach enables the production of high-density SiC/SiC composites (>3.0 g/cm^3^) but requires precise control of sintering conditions to minimize residual porosity (<5%) and prevent fiber degradation above 1900 °C. According to several authors [96,98,99,100,101,102], sintering is a fundamental technique for densifying ceramic matrices, providing flexural strength of 400–600 MPa and creep resistance at >1300 °C. Various process modifications have been reported in the literature, including hot isostatic and reactive sintering, the use of active fillers (e.g., Al, B, Y_2_O_3_) to reduce temperature by 100–200 °C and improve wettability, as well as advanced methods such as spark plasma sintering (SPS), which minimizes grain growth and enhances mechanical properties, achieving near-theoretical density in minutes. The main advantages of sintering are high density and low porosity, versatility for different ceramic systems, and improved strength, thermal resistance, and durability of the composite. Disadvantages include the need for high temperatures, risk of fiber damage, shrinkage deformations during the fabrication of large components, and the high cost of specialized equipment.

The matrix fabrication methods for SiC/SiC composites-CVI, PIP, LPI, and sintering in various modifications-represent a complementary range of technologies that allow control over microstructure, density, and material properties depending on the requirements of the intended application. CVI provides high-quality interphase boundaries and minimal thermal damage to the reinforcing fibers but is characterized by long processing times. PIP technology offers flexibility in design and the possibility of modifying the chemical composition of the matrix, yet it requires multiple processing cycles to achieve low residual porosity. LPI enables high productivity and applicability to large-scale components with complex reinforcement architectures, although limited microstructural control and the risk of residual free silicon remain technological challenges. Sintering methods, including hot pressing, hot isostatic pressing, reactive, and spark plasma sintering, yield dense and strong matrices but require high temperatures and may lead to degradation of the reinforcing phase. The selection and combination of these technologies, taking into account their advantages and limitations, are critical for the development of next-generation high-temperature SiC/SiC composites for aerospace and energy applications.

The next stage in evaluating the technological feasibility of the selected fabrication routes involves analyzing mechanical performance data harmonized across multiple testing conditions. Table 3 summarizes representative mechanical properties of SiC/SiC composites, annotated with the testing atmosphere, exposure regime, and process route. An additional “fiber/interface” column is included to account for the anisotropy associated with fiber architecture and interphase design, which play a decisive role in mechanical response and oxidation resistance.

Table 3 summarizes the mechanical performance of SiC/SiC composites, emphasizing the influence of reinforcement architecture, fiber type, and densification method on overall strength and fracture behavior. The results show that optimized fiber–matrix interfaces, high-quality densification (CVI, PIP, LSI), and reduced porosity significantly enhance mechanical stability and damage tolerance. Variations among fiber grades (e.g., Nicalon, Hi-Nicalon, Tyranno) mainly affect the strength and elastic response through differences in microstructure and bonding efficiency. Fracture toughness and fatigue resistance are governed by interphase composition and residual stress relaxation, confirming the importance of interface engineering for reliable performance under cyclic and high-temperature loading. Collectively, these observations demonstrate that the balance between fiber reinforcement, matrix consolidation, and interphase design defines the mechanical robustness and long-term durability of SiC/SiC composites for nozzle applications.

Current applications of SiC/SiC composites confirm the maturity of the technologies for hot-section engine components. In aerospace engineering, both oxide and non-oxide composites are implemented in combustion chambers/combustors and exhaust nozzles [84]; these components are the closest analogs to our nozzle assemblies. Concurrently, the automotive industry has accumulated extensive experience with SiC-SiC brake disks, including serial implementation (e.g., in the Porsche 911 Turbo and Mercedes-Benz CL 55 AMG F1), achieving a weight reduction of ~30 kg per vehicle while maintaining service life under high thermal loads [99]. This “tribological” case is particularly relevant for nozzle materials, demonstrating the simultaneous high thermal resistance, wear, and impact durability of SiC-SiC under cyclic thermo-mechanical loading.

For hot-flow applications, where long-term thermal stability is critical, technological solutions based on SiCf/SiC (PIP) have proven effective: pre-treatment of SiC fillers and strict atmospheric control reduce matrix degradation; the loss in flexural strength after 1400 °C was only ~15.3%, directly indicating suitability for prolonged exposure to hot gas flows [95]. Comprehensive reviews of SiC/SiC composites report a wide range of aerospace applications-from hot-section turbine components and thermal protection panels to rocket propulsion assemblies-highlighting continuously reinforced SiC/SiC and C/SiC as the baseline architectures for high-temperature components [98]. Collectively, these data justify the selection of continuously reinforced SiC/SiC and C/SiC as reference materials for nozzle blocks and provide benchmarks for thermal and fatigue performance requirements.

### 2.4. Metal Matrix Composites (MMC)

Metal matrix composites (MMCs) are high-performance structural materials optimized for operation under intense heat flux (typically 5–20 MW·m^−2^) and cyclic thermo-mechanical loading. The combination of a metallic matrix (Cu-, Ni-, Ti-, Al-, Mo-, and W-based systems) with dispersed and/or continuous reinforcing phases (carbon fibers, SiC, Al_2_O_3_, TiC, TiB_2_, B_4_C, diamond, usually 10–60 vol.%) allows targeted adjustment of thermal conductivity (ranging from 150 to 400 W·m^−1^·K^−1^ depending on system), elastic modulus (70–400 GPa), and coefficient of thermal expansion (CTE, 4–18 × 10^−6^ K^−1^), matching them to interfacing nozzle components and coatings. For components exposed to high heat fluxes, Cf/Cu-, W-Cu-, and Ni-based systems are preferred, providing high thermal conductivity (up to ~300 W·m^−1^·K^−1^ for Cf/Cu, 170–220 W·m^−1^·K^−1^ for W-Cu) along with sufficient high-temperature strength and impact toughness. To reduce interfacial diffusion and oxidative degradation, barrier layers (1–10 µm of Cr, Nb, Mo) and high-temperature coatings (Re, W, HfC, SiC), including functionally graded architectures with gradual composition transitions over 100–500 µm, are employed [32,33,114].

The architecture of MMCs is classified according to the form and topology of reinforcement: continuous (1D UD layups, 2D woven patterns-plain/satin/twill, angled ±θ; 3D orthogonal/stitching/braided preforms) and discrete (short fibers 100–500 µm, whiskers < 5 µm diameter, particles 50 nm–50 µm), see Figure 3a,b. Textbooks and review sources emphasize that discretely reinforced MMCs dominate industrial practice due to their manufacturability and quasi-isotropic properties. In contrast, continuous fiber reinforcement is preferred when directional stiffness (E_1_ up to 400 GPa) or strength (σ_1_ > 1 GPa) is required and allows pre-weaving or braiding of preforms prior to metal infiltration, which enhances interlaminar strength by 20–40% but complicates fabrication [114,115,116].

A distinct class comprises interpenetrating phase composites (IPC), in which the metallic phase forms percolating 3D networks with reinforcement fractions of 30–50%. Over the past decade, interest in IPC-MMCs has grown significantly: they offer unique combinations of thermal conductivity (150–250 W·m^−1^·K^−1^), stiffness (100–250 GPa), and thermal expansion matching (6–10 × 10^−6^ K^−1^) for thermally stressed components, although they remain more technologically challenging to manufacture due to processing times 2–3× longer than for conventional MMCs [32]. Applied reviews also note a trend toward hybrid architectures (combining fibers, particles, and nanophases), which are used to achieve directional strength while maintaining quasi-isotropic thermal response in high-heat regions [114,116].

**Figure 7:**
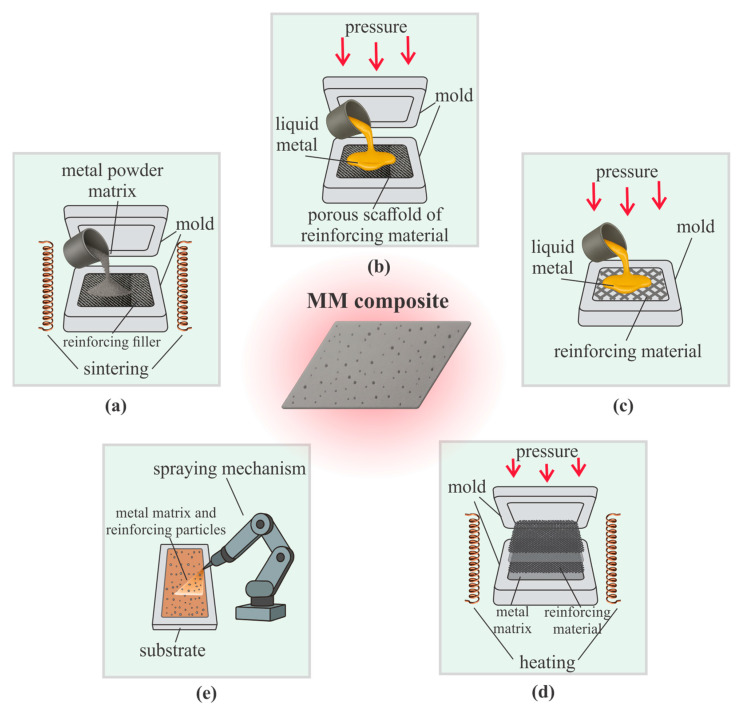
MMC fabrication schemes: (**a**) powder metallurgy, (**b**) liquid infiltration, (**c**) casting, (**d**) diffusion bonding, (**e**) spray deposition.

The matching of thermo-mechanical properties between the matrix and the reinforcing phase also plays a crucial role, directly influencing the choice of MMC fabrication technology. In practice, the most common routes are powder metallurgy, liquid-phase infiltration (including under pressure), and casting; in more specialized cases, diffusion bonding and spray deposition are employed. The corresponding schemes are shown in Figure 7 with proper adaptation, these approaches can also be applied to ceramic composites.

Powder metallurgy (Figure 7a). Based on mixing metallic powders (<10–50 µm) with a reinforcing phase (5–40 vol.%), followed by pressing at 200–800 MPa and sintering at 60–90% of the melting point. This approach helps prevent segregation and undesirable chemical reactions at the interface [117]. Studies [30,118] note that powder metallurgy enables fine-grained structures (<5 µm grain size), high relative density (>95%), and in situ synthesis of phases during sintering, enhancing thermal stability and fracture resistance. Research including [31,119], demonstrated that for titanium matrices, controlling powder size (10–20 µm) and sintering conditions (1200–1350 °C, 1–3 h) allows uniform distribution of SiC or carbon nanotubes, improving strength by 20–40% and toughness by 15–25%. Authors [117,120] emphasize versatility: adjustments to pressing pressure, mechanical alloying time (5–20 h), and sintering cycles can control porosity (2–10%), microstructure, and reinforcement distribution.

Liquid-phase infiltration (Figure 7b). A porous ceramic or fiber preform (30–60% porosity) is impregnated with a molten metal to form a dense, highly reinforced structure [121]. The process can occur by capillary action or under pressure; squeeze infiltration at 50–150 MPa provides >98% density and a reliable interface [34,121,122]. Modifications include vacuum, gas, and mechanical infiltration, with the choice of mode depending on the viscosity of the molten metal and wettability of the reinforcement [121]. Variable pressure infiltration (cyclic loading between 50–120 MPa) improves pore filling efficiency by 10–20% and reduces defects, especially in fiber-reinforced composites [123]. In W-Cu systems, applied pressure determines the distribution of Cu (typically 30–40 vol.%) and overall flexural strength (up to 200–250 MPa). For Al/SiC, densities of >99% and uniform microstructures with particle spacing < 5 µm have been achieved [124]. Reviews confirm the versatility of the method, highlighting that precise control of pressure, temperature, and wettability makes it promising for highly stressed components, including nozzle assemblies [121,125].

Casting (Figure 7c). This method is based on introducing the reinforcing phase into molten metal at 600–1700 °C followed by solidification, making it one of the most widely used and cost-effective approaches for producing MMCs. Stir casting has been extensively studied in [28,126,127,128,129], with emphasis on optimizing melt temperature (±20 °C around liquidus point), stirring speed, and stirring time (5–20 min) together with the addition of modifiers (e.g., Mg, TiB_2_ up to 1–3 wt.%) to improve wettability and prevent particle agglomeration. These measures allow for more uniform distribution of SiC and other reinforcements, although challenges with porosity (2–8%) and defect formation remain. Conversely, studies [130,131,132] explored squeeze casting, where the melt is injected under pressure (50–150 MPa) into a mold or preform, significantly reducing shrinkage defects (by 30–50%) and producing a denser composite structure with relative densities > 98%. These investigations demonstrated that applied pressure improves matrix-reinforcement contact, resulting in materials with 15–30% higher tensile strength and improved wear resistance compared to conventional casting.

Diffusion bonding (Figure 7d). This method is based on solid-state joining at elevated temperature (0.6–0.8 T_m_, i.e., typically 500–1100 °C) and pressure (5–50 MPa) for holding times of 0.5–3 h, where the bond forms through mutual atomic diffusion without melting the matrix. Researchers have applied this method to various systems: study [133] showed that adding an Al-Li interlayer (10–20 µm thickness) in 6061/Al_2_O_3_ aluminum composites improves tensile strength by 20–25% by breaking the oxide film; study [134] analyzed interface evolution and the effect of interlayers on joint strength by up to 30%; in [135] multilayer composite tools reinforced with WC were fabricated via diffusion bonding of hot-pressed preforms at 900–1000 °C, 20 MPa. Study [136] demonstrated successful application of the method for joining titanium and aluminum alloys in aerospace structures, achieving bond strengths of >200 MPa. These studies confirm that diffusion bonding is a versatile approach, enabling reliable and homogeneous joints across a wide range of MMC systems.

Spray deposition (Figure 7e). This technique involves propelling metallic powders (10–50 µm) in a high-velocity gas stream at 200–600 m/s, allowing them to deposit onto a substrate with minimal thermal impact. Its main advantage is the formation of dense coatings and composite structures while preserving the reinforcing particle architecture. Study [137] demonstrated cold-sprayed aluminum and Al/Cu coatings on polymer matrices with carbon fibers, achieving strong adhesion (>60 MPa) and enhanced functional properties. Reference [138] employed plasma spraying of spray-dried Al-Si powders with carbon nanotubes (CNTs, 0.5–2 wt.%), obtaining uniform CNT distribution and significant increases in strength (+25–40%) and elastic modulus (+20–30%) according to nanoindentation data. Study [139] showed that cold spray can produce aluminum-alumina composites, where dense coatings (>95% density) form through intense plastic deformation of particles. Reference [115] noted that spray deposition enables precise control of layer thickness (50 µm-several mm) and morphology. In ref. [140] noted that spray deposition enables precise control of layer thickness (50 µm-several mm) and morphology. In ref. [137] also emphasized that cold spraying minimizes defects typical of thermal spraying, such as oxides and residual stresses, and can be successfully applied for metallization of polymeric and ceramic substrates.

The reviewed technological approaches for producing MMCs allow tailoring their structure and achieving the desired operational properties. However, to assess the effectiveness of these materials in rocket engine components, their mechanical properties are of fundamental importance. Typical values are: tensile strength 200–600 MPa (Al/SiC, Ti/SiC, Ni/Al_2_O_3_), flexural strength 300–800 MPa, fracture toughness 10–25 MPa·m^1/2^, elastic modulus 100–400 GPa, hardness 100–300 HV, depending on matrix and reinforcement [30,31,32,114,115,116]. These combinations determine reliability under static and cyclic loads, as well as at elevated temperatures. Table 4 presents average values of MMC properties from the literature.

Table 4 summarizes the mechanical characteristics of metal matrix composites (MMCs) and highlights the strong dependence of their properties on matrix type, reinforcement nature, and fabrication route. The data indicate that Ti- and Nb-based MMCs generally provide superior strength and stiffness, while Al-based systems offer lower density and better manufacturability. The efficiency of reinforcement is largely governed by particle or fiber distribution and the strength of interfacial bonding, which are controlled by processing methods such as powder metallurgy, infiltration, or diffusion bonding. Systems with higher ceramic content demonstrate increased stiffness and compressive strength but reduced fracture toughness, underscoring the trade-off between rigidity and ductility typical for MMCs. Overall, the dataset confirms that the optimization of reinforcement morphology and interfacial cohesion is essential for achieving a balanced combination of mechanical reliability and thermal stability in MMC components for nozzle assemblies.

In practical solid and hybrid rocket engines, W-based MMC throat inserts have been used and continue to be employed for decades. Historically, this was demonstrated in [145]: where the development, fabrication, and hot-fire testing of silver-infiltrated porous tungsten (W-Ag) throat inserts encapsulated in a solid W shell were carried out specifically as rocket nozzle throat inserts. These tests confirmed the manufacturability of encapsulation/venting and the erosion resistance under real nozzle conditions. Modern hybrid rocket engines utilize multi-interface nozzles with a combination of materials tailored to local thermomechanical loads. For example, ref. [146] reports the design and full-scale testing of a nozzle featuring a carbon–ceramic “cap” at the throat section combined with a copper-infiltrated tungsten (W-Cu) insert. It was shown that W-Cu provides the required high-temperature and corrosion resistance during prolonged operation, and the wear profile after a 200 s hot-fire test closely matched thermochemical erosion predictions. This directly confirms the applicability of W-Cu inserts specifically for the throat. Industry data is supported by governmental publications: DRDO (India) explicitly reports the use of W-Cu throat inserts and throat nozzles in rocket technology, alongside other components such as jet vanes, indicating serial production [147]. Beyond W-Ag/W-Cu for the throat, high-thermal-conductivity Cu-based MMCs are currently considered for nozzle-side liners in liquid rocket engine assemblies. In ref. [148] the development and fabrication of subscale chamber liners from Cu-NARloy-Z alloy reinforced with diamond (Cu-diamond, NARloy-Z-D) via Field Assisted Sintering (FAS/SPS) with subsequent diffusion welding of ring sections is presented. The authors demonstrate achievable effective thermal conductivity exceeding standard Cu liners (NARloy-Z, GRCop-84) and justify the potential of such MMC liners to increase maximum heat flux and hot-section lifetime. This is directly relevant to the transition region toward the nozzle and the nozzle liner. NASA historical reports from the late 1960s [149] also include hot-fire test results of nozzles with perforated/infiltrated W-Ag/W inserts, showing minimal throat erosion and exploring liner design variants. Collectively, these sources confirm the long technological trajectory of MMCs in rocket engine throat inserts.

### 2.5. Ablative Composites

Ablative composites are multi-component materials in which a polymer matrix-primarily phenol-formaldehyde, epoxy, or polyimide resins-is reinforced with fibrous fillers such as carbon, aramid, basalt, or glass fibers. Typically, the resin content is 40–55 wt.%, while fiber reinforcement constitutes 45–60 wt.% of the composite. Additionally, dispersed additives (oxides, nitrides, carbides) are introduced in amounts of 5–15 wt.% to enhance thermal resistance and regulate the ablation rate. Under extreme heat fluxes of up to 3–5 MW·m^−2^, the organic matrix carbonizes, forming a thermally insulating carbon layer, while the released gases create a barrier that reduces heat transfer to underlying structures. Through the combination of these mechanisms, ablative materials exhibit high thermal resistance (up to 2000 °C in oxidative environments and 2500 °C in inert atmosphere), low thermal conductivity (0.2–0.4 W·m^−1^·K^−1^), and durability under aerodynamic heating. These properties make them indispensable in aerospace applications, particularly for thermal protection systems and rocket engine nozzle components operating under intense thermal and mechanical loads [150,151].

The greatest advantages in thermal protection of nozzle assemblies have been demonstrated by 3D and 4D architectures, which reduce property anisotropy and ensure uniform maintenance of nozzle geometry, whereas 1D/2D structures show lower ablation resistance (Figure 3a) [152,153,154]. Plasma and oxy-acetylene tests with heat fluxes of 2–4 MW·m^−2^ indicate that fiber orientation governs not only the linear recession rate (typically 0.05–0.20 mm/s under short-duration laboratory exposures) but also the morphology of the residual surface, values which should be interpreted as indicative test data rather than universal operational benchmarks [153,154]. For three-dimensional needled and pierced textures, a key parameter is the rate of pore “sealing” by carbide formation and oxidation products: the higher this rate, the lower the ablation rate. In this context, chopped-web needled structures demonstrate mass ablation rates lower by approximately 10–20% relative to fine-weave pierced ones due to the faster formation of the protective layer [3]. Overall, dense 3D/4D-reinforced preforms provide uniform erosion and low surface roughness, while fiber orientation and the nature of the pore structure directly determine the depth of thermal damage (1–3 mm typical for short-duration plasma tests) and the durability of nozzle components (Figure 3b,c) [152,153,154].

**Figure 8:**
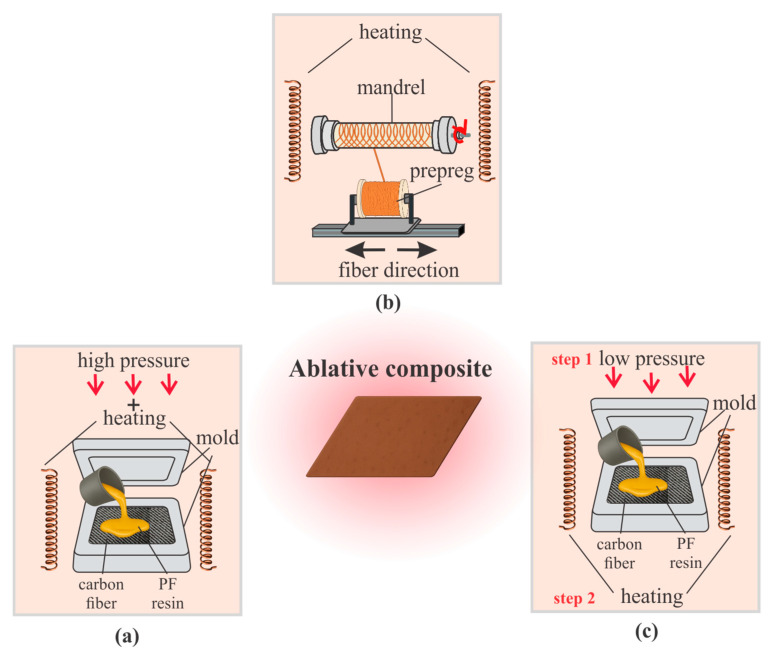
Main manufacturing methods for ablative composites: (**a**) compression molding; (**b**) filament winding; (**c**) 3D forming followed by sintering or vulcanization.

The choice of reinforcement architecture in ablative composites is determined by service life (50–200 s operation in SRM nozzles), allowable mass loss (<5–10% of the initial weight), and heat transfer conditions. An optimal combination of matrix, fiber, and reinforcement scheme ensures resistance to extreme loads. Figure 8 illustrates the main manufacturing routes: compression molding, winding, and 3D forming with sintering or vulcanization.

Figure 8a shows the compression molding process, in which reinforced preforms are shaped in a closed mold under heat (150–200 °C) and high pressure (5–20 MPa), with a molding duration of approximately 15 min, which corresponds to typical parameters reported for the fabrication of silicone-rubber-based ablative composites ensuring dense packing of fibers and uniform curing of the matrix. Historically, this method has been used for phenol-formaldehyde and epoxy resins with various fillers and remains fundamental in the production of thermal protection elements [151]. It provides low porosity (<5%), reproducibility, and uniform distribution of reinforcing phases, which is critical for ablation resistance [155]. Tests confirm the formation of a stable carbon layer and recession rates in the range of 0.1–0.15 mm/s under short-duration plasma torch exposures, while increased density (1.6–1.8 g/cm^3^) and optimized architecture further reduce linear and mass ablation rates [152,156].

Figure 8b illustrates the filament winding process, in which resin-impregnated fibers (carbon, glass, silica, or aramid) are wound onto a rotating mandrel under controlled angle (±15–60°) and tension. This method enables the fabrication of cylindrical and conical nozzle elements with high precision (dimensional tolerance ±0.2–0.5 mm) and uniform structure. Studies confirm its effectiveness: Ref. [157] demonstrated that optimizing fiber angles improves erosion resistance by 15–25%; Ref. [158] identified differences in ablation behavior between carbon- and silica-phenolic composites, with linear recession rates of 0.05–0.12 mm/s and 0.08–0.18 mm/s, respectively, reported in short-term oxy-acetylene tests; Ref. [159] highlighted the importance of controlling pressure and tension; Ref. [160] emphasized the need for precise mechanical finishing. Filament winding combines controlled fiber orientation with the capability to produce large components (diameter up to 2–3 m), making it a key technique for manufacturing ablation-resistant composites [161].

The 3D forming method followed by sintering or vulcanization, shown in Figure 8c, represents one of the modern technological strategies for producing ablative composites, enabling the implementation of spatial reinforcement architectures with high resistance to thermomechanical loads. Review [162] indicates that this approach is considered alongside CVI, PIP, and resin transfer molding as a promising matrix consolidation method for carbon-phenolic materials. Study [163] describes the fabrication of 3D-woven C/Ph composites, subsequently impregnated with phenolic resin and thermally cured, which reduces delamination by 30–40% and enhances ablation resistance. Authors in [19] demonstrated that the use of three-dimensional architectures produced by this method significantly improves thermal stability (up to 2000 °C) and ablation resistance during high-temperature testing. Furthermore, research on elastomeric systems notes that a similar scheme-forming a 3D preform followed by matrix vulcanization-allows for uniform stress distribution and increases component reliability under intense thermal flux (>4 MW·m^−2^).

The compression molding method provides simplicity and reliability, filament winding ensures precise fiber orientation and enables the fabrication of large-scale components, while 3D forming allows for spatial architectures with enhanced resistance to delamination and thermomechanical loads. For a generalized assessment of the properties of ablative materials in nozzle assemblies, Table 5 presents the averaged values of their main mechanical characteristics.

Table 5 summarizes the mechanical and thermal performance of ablative composites used for thermal protection applications. The results show that the introduction of carbon fibers into phenolic matrices markedly enhances strength, toughness, and fatigue resistance compared with unreinforced or quartz-based systems. Fiber reinforcement mitigates brittle fracture and maintains structural integrity during high thermal gradients and dynamic loads. The mechanical response of these materials strongly depends on fiber volume fraction, orientation, and post-curing pyrolysis behavior, which defines both modulus and residual strength at elevated temperatures. The formation of a stable carbonized char layer ensures continuous thermal protection up to 2000–3000 °C, while the fibrous framework prevents crack propagation and strength loss under cyclic loading. Overall, the dataset confirms that fiber-reinforced phenolic composites achieve an optimal balance of lightweight structure, thermal stability, and damage tolerance, making them the most efficient materials for single-use nozzle liners.

Ablative composites, primarily carbon- and phenolic-based systems, remain key materials for nozzle blocks. NASA reports note that the linings of solid-propellant rocket engine nozzles are made from carbon-phenolic composites, where a char layer forms during operation, absorbing heat and protecting the structure [170]. Arc-jet tests show that the endothermic pyrolysis of the phenolic matrix and gas release effectively reduce heat transfer, maintaining material integrity at temperatures above 2000 °C [170]. Recent reviews confirm the widespread use of such composites in thermal barriers and nozzle blocks, highlighting their high specific strength and thermal stability [162]. Specifically, work [162] emphasizes the effectiveness of carbon-phenolic systems in rocket engine nozzles, while [170] demonstrates their successful application in tests for surface-to-air class engines. Recent studies [171] point to the potential of nanostructured phenolic composites and aerogel-modified systems, providing enhanced thermal resistance and reduced ablation rates. Altogether, this confirms the relevance of ablative composites for nozzle blocks and the directions for their further development.

## 3. Comparative Analysis of Composites for Nozzle Assemblies

Based on the averaged properties and processing routes presented for five classes of composite materials (C/C, C/SiC, SiC/SiC, metal matrix systems, ablative composites), final conclusions can be drawn regarding the rational allocation of materials within uncooled nozzle assemblies and the identification of the “core” structural material. Each class possesses a unique combination of thermal resistance, strength, and manufacturability, determined by the nature of the matrix and fibrous reinforcement. C/C composites provide exceptional thermal resistance (stable at T > 2500 °C in an inert atmosphere) and maintain mechanical properties up to 1600–1800 °C in oxidizing environments when protective coatings are applied. They exhibit low thermal expansion and high resistance to thermal shocks, explaining their successful use in the hottest zones of nozzle structures. C/SiC composites are slightly inferior to C/C in absolute thermal resistance (suitable for operation up to 1300–1500 °C in oxidizing atmospheres) but show better oxidation stability due to the ceramic matrix. SiC/SiC composites demonstrate even higher heat resistance (>1600 °C) and nearly complete oxidation immunity because both fibers and matrix are silicon carbide. They also feature high elastic modulus and strength, but require expensive continuous SiC fibers and complex fabrication processes, limiting widespread industrial use. Metal matrix composites combine properties depending on the choice of metallic matrix and reinforcing phase: they can operate at 800–1000 °C, offering high thermal conductivity and impact toughness thanks to the metallic component. However, at higher temperatures, the matrix softens and oxidizes. Ablative composites based on polymer matrices do not achieve the same mechanical strength at ambient conditions but, under extreme thermal flux, form a protective char layer that efficiently shields the structure and dissipates heat. Controlled surface ablation allows them to withstand brief exposure to gas temperatures exceeding 2000 °C while preserving nozzle integrity throughout engine operation.

Table 6 provides a comparative overview of the analyzed composite classes, integrating their mechanical reliability, environmental stability, and manufacturability aspects. The data clearly demonstrate that carbon-based composites (C/C, C/SiC, SiC/SiC) remain the core materials for non-cooled nozzle assemblies due to their combination of high thermal resistance, structural strength, and oxidation control through tailored interphases. In contrast, metal matrix composites, despite their superior static strength, are constrained by oxidation sensitivity and high density, restricting their use to auxiliary elements. Ablative composites occupy a complementary niche, offering reliable one-time thermal protection through controlled surface consumption and ease of large-scale fabrication. Overall, the comparison highlights the trade-offs between strength, oxidation resistance, and manufacturability, guiding rational material selection for advanced nozzle architectures.

To extend the comparative framework beyond material performance, the analysis in Table 7a,b incorporates the manufacturing perspective, distinguishing between expendable and reusable non-cooled architectures. This classification links material selection with production cadence, scalability, and technology maturity, thereby connecting structural design logic with industrial feasibility.

The split comparison underscores the technological divergence between expendable and reusable non-cooled architectures. Expendable systems prioritize low-cost, high-throughput fabrication routes such as RS or molding, where rapid cycle time and broad material supply enable scalability. In contrast, reusable configurations rely on high-precision CVI-based or hybrid densification processes, where long processing cadence and stringent coating or interphase control govern overall feasibility. The TRL and supply maturity remain highest for ablative and C/C systems, while SiC/SiC and hybrid composites show increasing adoption potential driven by performance gains but constrained by production complexity. Collectively, this framework integrates material behavior with manufacturability and lifecycle considerations, providing a unified basis for decision-making in advanced nozzle design.

C/C composites represent the most balanced choice for non-cooled nozzles, combining high thermal resistance, sufficient strength and impact toughness, and good thermal cycling durability. Their practical applicability has been demonstrated in both flight and test examples: the RL10B-2 features an extendable C/C nozzle with protective coating, which passed qualification fire tests (AIAA 1997–2672; 1998–3363; 2002–3585) [172]. Composite nozzles have also been implemented in European cryogenic upper-stage engines, such as Vinci (AIAA 2005–3757) [173]. or throat inserts in solid-propellant and some liquid engines, graphite and C/C are traditionally used, as confirmed by studies on erosion and ablation of these materials in nozzles [174]. Long-duration fire tests of C/C nozzles in hydrogen-oxygen flames demonstrate their operability under radiative cooling (including without regenerative cooling), provided that oxidative protection is applied. To extend service life, C/C elements require effective anti-corrosion/oxidation barriers (e.g., SiC coatings), as reflected in the RL10B-2 qualification programs and subsequent developments [3,175].

Other classes of composites are best applied selectively across nozzle regions, according to their advantages. C/SiC composites are suitable for expansion sections and walls, where mass reduction and ablation resistance under moderate thermal loads are important; they have been successfully used in the Vinci nozzle. Throat inserts are also possible in non-aggressive environments or with protective coatings, providing longer service life than pure carbon materials. SiC/SiC composites are optimal for reusable and long-duration systems in hot zones (throat, inner lining) without cooling; operability has been demonstrated, for example, in the X-33 program. However, high cost, brittleness, and limited statistical data necessitate careful non-destructive testing and precise qualification. MMCs should be used selectively where thermal conductivity and impact strength are critical: throat heat management can be achieved with Cf/Cu or W-Cu inserts that conduct or dissipate heat, at the expense of mass. Refractory-matrix composites (Ni, Nb) are appropriate for fasteners and external casings at T ≤ 800–1000 °C; they require oxidation protection, and their production technologies are less mature than those for C/C or ablatives. Ablative materials remain a rational choice for single-use solid rocket motors and highly loaded nozzles: they provide thermal protection via controlled surface consumption, and simple forming techniques (pressing, winding) allow the manufacture of large-scale linings. The limitation is non-reusability; nevertheless, for single-use or low-service-life systems, ablatives offer the most cost-effective solution, additionally benefiting from low density and resistance to thermal shock.

Building upon the material specific characteristics outlined above, a comparative assessment is required to identify the most rational combinations of composites within a single nozzle assembly. Such integration allows translating isolated material data into system-level design logic, where performance, manufacturability, and durability can be balanced according to operational conditions.

Considering operational and manufacturing aspects, the optimal configuration of a non-cooled nozzle employes a combination of several composite classes. C/C is best used in the hottest region-the throat-where ultra-high thermal resistance and thermal shock endurance are critical. In the diffuser segments, which experience lower temperatures but cover larger areas, lighter C/SiC or C/C-SiC hybrid composites are appropriate, providing sufficient heat resistance along with improved oxidation stability. The structural casing and attachment elements, located outside direct gas exposure, can be made from metal matrix composites or conventional materials offering the required strength and matched coefficient of thermal expansion. When an ablative liner is present, the outer casing may use lower-temperature materials (e.g., carbon-fiber composites, aluminum), as the ablative layer reliably shields against heat. Ultimately, C/C remains the baseline choice for the most thermally loaded load-bearing parts, while C/SiC and SiC/SiC are suitable for expansion and secondary regions where oxidation resistance and cyclic durability are important; ablative materials are indispensable in single-use designs and for simplified manufacturing.

Modern non-cooled nozzles are designed according to the principles of multifunctional materials engineering: different sections are made from composites optimized for their specific conditions to maximize reliability, durability, and specific impulse efficiency. Reviews confirm that C/C currently represents the most balanced solution; further advancements-such as nanomodification, hybrid architectures, and diffusion barriers-along with improvements in non-destructive testing and scalable manufacturing, will enhance the service life and safety of all composite classes. The integrated use of their respective advantages paves the way for lightweight, resilient nozzles capable of operating near extreme limits while maintaining the required reliability.

To translate the above comparative analysis into a form directly applicable to engineering practice, a concise operational framework is proposed (Table 8).

It integrates three critical parameters-nozzle zone, environment, and mission type (expendable or reusable)-and provides corresponding recommendations for material class, densification route, and protective coating/interface to support rapid decision-making during nozzle design.

The presented matrix establishes a direct relationship between operational parameters and material solutions, serving as a quick-reference tool for engineers.

To demonstrate its practical applicability, two representative cases are outlined below.

*Case A-Expendable Solid Rocket Motor.* A tactical solid rocket motor (~60 s burn, fuel-rich gases, ≈2800 °C) represents a typical single-use configuration that includes the throat, convergent section, and outer casing. The throat is made of C/C composite fabricated via PIP without coating, while the convergent section employs a C/SiC composite obtained through reactive sintering (RS) with a SiO_2_ glassy sealant. The outer casing consists of a carbon-phenolic ablative liner produced by compression molding. This configuration provides a lightweight ablative-carbon architecture with predictable erosion behavior and full thermal protection during single-use operation.

*Case B-Reusable Upper-Stage Engine.* A reusable orbital-insertion engine (multi-burn mode, oxidizing environment, ≈3000 °C) comprises the throat, divergent section, and support liner. The throat utilizes a C/C composite manufactured via a hybrid CVI + PIP route with a SiC coating, while the divergent section applies a SiC/SiC composite densified by CVI with a BN interphase and SiC top coat. The support liner employs a W-Cu MMC produced by diffusion bonding and protected by a Mo overlay. This multimatrix design sustains more than ten thermal cycles with minimal oxidation and erosion, demonstrating suitability for reusable propulsion systems.

This framework connects the comparative material database with practical design considerations, enabling balanced optimization of performance, mass, lifetime, and manufacturability in next-generation non-cooled rocket-nozzle assemblies.

To extend the comparative framework toward operational reliability, Table 9 summarizes the dominant degradation mechanisms for each major material family used in non-cooled nozzle assemblies. It integrates the principal thermo-mechanical and oxidative damage modes with corresponding mitigation strategies, non-destructive inspection (NDE) intervals, and coating or interphase lifetime assumptions. This mapping establishes a practical link between material degradation behavior and maintenance planning, bridging the gap between performance evaluation and operational durability.

As evident from Table 9, each composite family exhibits a characteristic degradation pathway governed by the interplay of temperature, oxidation kinetics, and mechanical stress. C/C and C/SiC are mainly affected by oxidation and thermal-shock-induced cracking, which can be mitigated through SiC- or HfB_2_-based coatings and hybrid densification routes such as CVI + PIP. SiC/SiC show superior oxidation resistance but rely on precise interphase engineering and periodic NDE to ensure coating integrity. Metal matrix composites are constrained by matrix oxidation and creep, while ablative materials degrade in a controlled manner, providing reliable single-use protection. Integrating this degradation-mode perspective into the material-selection framework strengthens the predictive capability for service lifetime and inspection scheduling in advanced non-cooled nozzle architectures.

Building upon the comparative analysis presented above, several critical research gaps and innovation prospects can be identified in the current development of multimatrix composite materials for rocket nozzle applications. Although significant progress has been achieved in the design, processing, and qualification of composite systems, their large-scale implementation and long-term reliability remain constrained by several unresolved factors.

First, most of the available materials are optimized for either thermal or mechanical performance, rarely providing an optimal combination of both. Carbon–carbon composites exhibit outstanding high-temperature capability but limited oxidation resistance, whereas SiC-based systems provide superior oxidative stability but suffer from brittleness and low fracture toughness. Second, manufacturing scalability and process efficiency remain challenging. Techniques such as chemical vapor infiltration and reactive sintering ensure excellent microstructural uniformity but involve long processing times, high energy consumption, and limited adaptability to complex geometries. Third, the understanding of multi-scale degradation mechanisms-especially interphase oxidation, fiber–matrix debonding, and fatigue under coupled thermo-mechanical loading-remains incomplete, limiting predictive lifetime modeling. Fourth, sustainability considerations have not yet been fully addressed. Environmental impacts associated with precursor synthesis, energy-intensive sintering, and end-of-life disposal of phenolic- or pitch-based composites are seldom quantified in current research.

Future innovation is expected to focus on several directions. Nano-modification and hybrid reinforcement strategies, involving carbon nanotubes, graphene, or ceramic nanoparticles, are anticipated to improve interfacial bonding, oxidation resistance, and damage tolerance. Multimatrix and functionally graded architectures, integrating polymeric, ceramic, and metallic matrices within a single nozzle assembly, could enable spatial tailoring of thermal and mechanical responses. Additive and digitally controlled manufacturing, including 3D-printed preforms and reactive infiltration with real-time monitoring, offer pathways toward scalable, repeatable production of complex geometries. Finally, sustainable processing routes, such as polymer-derived ceramics synthesized at reduced temperatures or recyclable precursors, represent an emerging field bridging high-performance materials with environmental responsibility.

Collectively, these research directions define the technological pathway toward next-generation multimatrix composite nozzles characterized by enhanced durability, reparability, and compliance with sustainability requirements for advanced propulsion systems.

## 4. Conclusions

Non-cooled rocket nozzles represent a technically justified design approach when the elimination of active cooling yields measurable gains in mass reduction, reliability, and structural simplicity. In such configurations, heat-loaded walls dissipate energy primarily through radiative transfer and controlled surface ablation, a strategy proven effective in single-use solid rocket boosters and large vacuum-stage engines. The material choice for non-cooled nozzle assemblies depends on heat flux intensity, thermal shock resistance, required lifetime, and environmental conditions (oxidizing or inert), as well as reusability requirements.

The comparative assessment conducted in this review highlights that carbon-based composites remain indispensable for the most thermally loaded throat regions due to their exceptional heat resistance (>2500 °C) and thermal shock tolerance. Silicon carbide-based systems (C/SiC and SiC/SiC) offer superior oxidation resistance and are optimal for expansion sections or reusable architectures. Metal matrix composites contribute mechanical strength and thermal conductivity but are limited to structural or support components exposed to moderate temperatures (<1000 °C). Ablative composites, typically carbon- or glass-fiber-reinforced phenolics, continue to provide the most efficient protection in single-use systems through controlled matrix pyrolysis and char formation.

Overall, the results confirm that eliminating active cooling is feasible when appropriate multimatrix combinations are employed to balance thermal, mechanical, and environmental performance. The proposed operational decision matrix (Table 8) establishes a practical framework linking nozzle zone, mission type, and material class, thereby supporting rational material selection in both expendable and reusable propulsion systems.

Future development should focus on hybridization and nano-modification strategies to enhance interfacial bonding, oxidation resistance, and reusability; on additive and digitally controlled manufacturing to improve reproducibility and scalability; and on sustainability-oriented routes, such as recyclable precursors and low-energy sintering, to minimize environmental impact. Collectively, these directions define the pathway toward next-generation multimatrix composite nozzles that combine durability, lightweight construction, and environmental responsibility.

## Figures and Tables

**Table 1:** Averaged Mechanical Properties of C/C Composites.

No.	Characteristic	Average Values	Atmosphere	Exposure	Process Route	Refs.
1	tensile strength	150–160 MPa	Inert (Ar)	Steady	PIP	[44,49,50,51]
2	flexural strength	170 MPa	Air	Transient	PIP	[22,42,47,50,51]
3	compressive strength	150–250 MPa	Air	Steady	CVI	[22,49,50,52]
4	impact strength/fracture toughness	5–10 MPa·m^1/2^	Inert (Ar)	Transient	CVI + PIP	[21,50,51,53]
5	elastic modulus (E)	~75 GPa	Air	Steady	CVI	[44,49,52,54,55,56]
6	high-temperature strength	~1750 °C	Air	Steady	LPI	[41,43,45,57,58,59]
7	fatigue strength	80–90 MPa, at 10^6^ cycles	Air	Cyclic	PIP	[41,51,53]

Note: All average data are presented as mean values; the uncertainty (±) corresponds to typical deviations reported in the cited references. Abbreviations: PIP-polymer infiltration and pyrolysis; CVI-chemical vapor infiltration; LPI-liquid polymer infiltration; Ar-argon.

**Table 2:** Mechanical properties of carbon–ceramic composites.

No.	Characteristic	Average Values	Atmosphere	Exposure	Process Route	Refs.
1	tensile strength	~230 MPa	Air	Steady	RS	[24,78,79,80]
2	flexural strength	290–300 MPa	Air	Transient	PIP	[78,79,80]
3	compressive strength	~390 MPa	Air	Steady	CVI	[74,78,79,80]
4	impact strength/fracture toughness	~5.45 MPa·m^1/2^	Inert (Ar)	Transient	CVI + PIP	[74,78,79,80]
5	elastic modulus (E)	~78.16 GPa	Air	Steady	RS	[64,68,79,80]
6	high-temperature strength	~88 MPa, at 1500–1700 °C	Air	Steady	CVI	[24,27,67,81]
7	fatigue strength	~164 MPa, at 10^6^ cycles	Air	Cyclic	RS	[66,68,80]

Note: All average data are presented as mean values; the uncertainty (±) corresponds to typical deviations reported in the cited references. Abbreviations: PIP-polymer infiltration and pyrolysis; CVI-chemical vapor infiltration; RS-reactive sintering.

**Table 3:** Average mechanical properties of SiC/SiC composites.

No.	Characteristic	Average Values	Atmosphere	Exposure	Process Route	Fiber/Interface	Refs.
1	tensile strength	~287 MPa	Air	Steady	CVI	2D plain weave/BN	[91,94,96,98,103]
2	flexural strength	~300 MPa	Inert (Ar)	Transient	PIP	3D braided/PyC	[86,92,97,98,104]
3	compressive strength	~232 MPa	Steam/moist air	Steady	LSI	2D plain weave/BN	[105,106]
4	impact strength/fracture toughness	~15 MPa·m^1/2^	Air	Steady	CVI + PIP	3D braided/BN	[86,88,96,107]
5	elastic modulus (E)	250–260 GPa	Air	Transient	CVI	2D woven/BN	[87,88,97,108]
6	high-temperature strength	1400–1600 °C	Air	Steady	CVI	3D braided/BN	[88,98,109,110]
7	fatigue strength	~133 MPa at 10^6^ cycles	Air	Cyclic	CVI	2D plain weave/PyC	[111,112,113]

Note: All average data are presented as mean values; the uncertainty (±) corresponds to typical deviations reported in the cited references. Abbreviations: PIP-polymer infiltration and pyrolysis; CVI-chemical vapor infiltration; LSI-liquid silicon infiltration; BN-boron nitride; PyC-pyrolytic carbon.

**Table 4:** Average mechanical properties of metal matrix composites.

No.	Characteristic	Matrix/Reinforcement	Average Values	Atmosphere	Exposure	Process Route	Refs.
1	tensile strength flexural strength	Al-SiC	~350 MPa	Air	Steady	Powder metallurgy	[30,129]
		Ti-6Al-4V	~1286 MPa	Inert (Ar)	Steady	Powder metallurgy	[31,102,119]
2	compressive strength impact strength/fracture toughness	Nb-SiC	~339 MPa	Air	Steady	Infiltration	[141]
		Ti-6Al-4V	~1968 MPa	Vacuum	Transient	Diffusion bonding	[142]
3	elastic modulus (E) high-temperature strength	Nb-SiC	~1785 MPa	Inert (Ar)	Steady	Hot pressing	[141]
		Ti-6Al-4V	~1320 MPa	Air	Cyclic	Diffusion bonding	[143,144]
4	fatigue strength characteristic	Nb-SiC	~ 15 ± 3 MPa·m^1/2^	Air	Steady	Casting	[35,119]
		Ti-6Al-4V	~ 22 ± 5 MPa·m^1/2^	Air	Steady	Powder metallurgy	[35]
5	tensile strength flexural strength	Nb-SiC	210–250 GPa	Inert (Ar)	Steady	Powder metallurgy	[35,114,118]
		Ti-6Al-4V	130–150 GPa	Air	Steady	Infiltration	[31,35,119]
6	compressive strength impact strength/fracture toughness	Al-SiC	300–350 °C	Vacuum	Transient	Diffusion bonding	[35,114]
		Ti-6Al-4V	~550 °C	Inert (Ar)	Steady	Hot pressing	[31,114,119]
7	elastic modulus (E)	Al-SiC	~195 MPa at 10^6^ cycles	Air	Cyclic	Diffusion bonding	[35,129,132]
		Ti-6Al-4V	~500 MPa at 10^6^ cycles	Air	Steady	Casting	[31,118,119]

Note: All average data are presented as mean values; the uncertainty (±) corresponds to typical deviations reported in the cited references.

**Table 5:** Averaged mechanical properties of ablative composites.

No.	Characteristic	Averaged Values	Atmosphere	Exposure	Process Route	Refs.
1	tensile strength	~60 MPa	Air	Transient	Compression molding	[38,110,164]
2	flexural strength	~90 MPa	Air	Transient	Filament winding	[38,164]
3	compressive strength	~150 MPa	Air	Transient	Hand lay-up	[110,165,166]
4	impact strength/fracture toughness	~50 kJ/m^2^ (from ~0.6 kJ/m^2^ to ~100 kJ/m^2^ with fiber)	Inert (Ar)	Steady	Compression molding	[167,168]
5	elastic modulus (E)	~20 GPa	Air	Transient	Compression molding	[168,169]
6	heat resistance (at high temperatures)	>1000 °C (formation of a carbon layer); to ~2000–3000 °C for C/C (in an inert environment)	Air/inert	Transient	Filament winding	[38,110,169]
7	fatigue strength	High	Air	Cyclic	Compression molding	[162]

Note: All average data are presented as mean values; the uncertainty (±) corresponds to typical deviations reported in the cited references.

**Table 6:** Comparative Table of Composites.

Composite Class	Mechanical Reliability	Environmental Stability	Mass-Dimensional Efficiency	Manufacturability and Cost-Effectiveness
C/C (Carbon–Carbon)	High: retains strength at T > 2500 °C; excellent thermal shock resistance.	Low without protection: carbon oxidizes at T > ~1500 °C (SiC coatings required); does not ablate (does not melt).	Excellent: very lightweight material (ρ ~1.8 g/cm^3^) with high specific strength.	Low: costly multi-step process (CVI/PIP); limited scalability and complexity for large components.
C/SiC (Carbon–Silicon Carbide)	High: strength comparable to C/C; operates up to ~1500 °C; good fatigue resistance under thermal cycling.	Enhanced: resistant to oxidation and erosion better than C/C; good ablative performance.	High: density lower than that of pure ceramics (~2.2 g/cm^3^); significant weight savings compared to metals.	Medium: requires infiltration techniques (CVI, LPI, PIP); reactive sintering accelerates fabrication; cost remains relatively high.
SiC/SiC (Silicon Carbide–Silicon Carbide, ceramic CMC)	Medium: high strength is maintained up to ~1600 °C; limited by ceramic brittleness, requires complex interfaces; thermal stability without degradation.	Excellent: practically non-oxidizing up to ~1400 °C; minimal ablation, high erosion resistance.	Moderate: density higher than carbon (~2.8 g/cm^3^); specific strength lower than C/C, but superior to metals.	Low: very expensive fibers and CVI/PIP processes; labor-intensive manufacturing with limited scalability; used only in specialized applications.
MMC (metal matrix composite)	Moderate: good strength and impact toughness up to 800–1000 °C; above this, load-bearing capacity decreases.	Low: the matrix is prone to oxidation/corrosion; barrier layers and coatings are required for high-temperature resistance; not designed for ablation (melts or vaporizes under extreme heat).	Low: density depends on the matrix (typically 3–9 g/cm^3^, over 2 × heavier than non-metallic composites); specific strength is lower than that of C/C and CMC.	High: relatively mature technologies (powder metallurgy, casting, etc.); suitable for serial production of large components; cost lower than C/C and CMC.
Ablative composite	Low: structural strength is limited (σ 60–100 MPa); sufficient for thermal protection functions but not for high mechanical loads.	Good (single-use): withstands heat fluxes > 2000 °C due to resin ablation; fully oxidizes over time (not suitable for multiple uses).	High: low density (~1.5 g/cm^3^); geometric stability maintained through uniform erosion (with 3D reinforcement).	Very high: technologically simple to manufacture (compression molding, winding); inexpensive raw materials and scalable production; cost-optimal for single-use systems.

Abbreviations: CVI—chemical vapor infiltration; PIP—polymer infiltration and pyrolysis; LPI—liquid polymer infiltration; Ceramic Matrix Composite.

**Table 7:** (**a**) Expendable, non-cooled architectures (indicative, single-use focus). (**b**) Reusable, non-cooled architectures (multi-cycle focus).

Material Class	Typical Nozzle Use	Preferred Route(s)	Cadence (Per Part)	Scale-Up Difficulty	TRL/Supply Maturity
(**a**)					
Ablative composites (C-Ph/Si-Ph)	Liners, outer walls, full blocks for SRM	Compression molding; filament winding	1–8 h molding/winding + 2–12 h cure	Low (large tools, simple lay-ups)	TRL 9/high (broad supply base)
Graphite/C/C (expendable throat)	Throat inserts in fuel-rich SRM/LRE	PIP (C/C); machined graphite	PIP 40–120 h per cycle × 3–6 cycles; graphite < 10 h machining	Medium (C/C porosity control)	TRL 8–9/medium-high
C/SiC	Convergent/expansion walls	RS (reactive sintering), PIP	RS 10–30 h; PIP 40–120 h/cycle	Medium (RS shrinkage control)	TRL 7–8/medium
(**b**)					
C/C (with coating)	Throat, close-to-throat rings	CVI + PIP hybrid; LPI	CVI 100–300 h + PIP 40–120 h/cycle (2–4 cycles)	High (densification & coating QA)	TRL 8–9/medium (coating supply is the bottleneck)
SiC/SiC	Divergent/convergent hot walls	CVI, PIP; CVI + PIP	CVI 120–300 h; PIP 40–120 h/cycle	High (interphase control, NDE)	TRL 6–8/medium (limited vendors)
C/SiC (hybrid)	Expansion walls, stiffened panels	RS; CVI/PIP hybrid	RS 10–30 h; CVI 120–300 h; PIP 40–120 h/cycle	Medium	TRL 7–8/medium

Abbreviations: SRM—solid rocket motor; LRE—liquid rocket engine; PIP—polymer infiltration and pyrolysis; CVI—chemical vapor infiltration; LPI—liquid polymer infiltration; RS—reactive sintering; NDE—nondestructive evaluation; QA—quality assurance; TRL—technology readiness level.

**Table 8:** Operational decision matrix for multimatrix composite nozzles.

Zone of Nozzle	Environment	Mission Type	Material	Manufacturing/Densification Route	Protective Coating/Interface	Notes/Typical Use
Throat	Oxidizing	Reusable	C/C or C/SiC	CVI + PIP	SiC or HfB_2_-SiC	Highest T > 2500 °C; requires oxidation barrier.
	Fuel-rich/inert	Expendable	Graphite or C/C	PIP	None	SRM applications; self-protective char layer.
Convergent section	Oxidizing	Reusable	SiC/SiC	CVI	BN + SiC	Stable under cyclic heating; moderate T (~1600 °C).
	Fuel-rich	Expendable	C/SiC	RS	SiO_2_ glassy sealant	Rapid and low-cost fabrication.
Divergent section	Vacuum/radiative	Reusable	SiC/SiC or MMC	RS/Powder metallurgy	SiC or W-based	High thermal conductivity; resists fatigue.
Outer casing	Air-cooled	Expendable	Ablative (C-Ph/Si-Ph)	Compression molding/filament winding	None	Lightweight; single-use thermal shield.
	Air-cooled	Reusable	MMC (Cu/W-Cu)	Infiltration/diffusion bonding	Mo or Re overlay	Structural integrity; multi-cycle durability.

Abbreviations: CVI—chemical vapor infiltration; PIP—polymer infiltration and pyrolysis; RS—reactive sintering; BN—boron nitride; Mo—molybdenum; Re—rhenium; SRM—solid rocket motor; MMC—Metal Matrix Composite.

**Table 9:** Dominant degradation mechanisms, mitigation strategies, and NDE (inspection) windows for major material families.

Material Family	Dominant Degradation Mode	Coupled Effects (Thermo-Shock/Oxidation/Erosion)	Mitigation/Design Strategy	NDE & Inspection Window	Coating/Interphase Lifetime Assumption	Material Family
C/C	Oxidation of carbon matrix-mass loss	High under O_2_-rich flow; aggravated by cyclic heating	Apply SiC or HfB_2_-SiC barrier; minimize oxygen ingress	Visual + ultrasonic (cycle 0–5)	SiC coating 5–10 cycles in oxidizing env.	C/C
C/SiC	Matrix microcracking and SiO_2_ volatilization	Moderate thermo-shock, mild erosion	Use glassy SiO_2_ self-healing + RS densification	IR thermography after 3–5 cycles	Coating integrity ~15 cycles	C/SiC
SiC/SiC	Fiber/matrix debonding, oxidation of BN interphase	Coupled thermal gradient and O_2_ diffusion	Multilayer BN/SiC interphase; dense CVI	Acoustic emission & CT (5–10 cycles)	20–30 cycles before recoat	SiC/SiC
MMC (Cu/W base)	Oxidation and creep of metal matrix	Limited to moderate heat flux (<1000 °C)	Mo or Re overlays, diffusion barrier	Eddy current, microcrack scan (annual)	Overlay reapply each 10–15 cycles	MMC (Cu/W base)
Ablative	Controlled matrix carbonization and surface recession	Erosion dominates; oxidation secondary	Maintain char continuity, filler loading 45–60 wt.%	Post-burn profilometry	Single-use (no coating)	Ablative

## Data Availability

The original contributions presented in this study are included in the article. Further inquiries can be directed to the corresponding authors.

## Abbreviations

The following abbreviations are used in this manuscript:

Abbreviation	Full form
C/C	Carbon–Carbon composite
C/SiC	Carbon–Silicon Carbide composite
SiC/SiC	Silicon Carbide–Silicon Carbide composite
MMC	Metal Matrix Composite
PIP	Polymer Infiltration and Pyrolysis
CVI	Chemical Vapor Infiltration
LPI	Liquid Polymer Infiltration
RS	Reactive Sintering
UHTC	Ultra-High-Temperature Ceramic
NDE	Non-Destructive Evaluation
TRL	Technology Readiness Level
EBC	Environmental Barrier Coating
